# The Prospect of Harnessing the Microbiome to Improve Immunotherapeutic Response in Pancreatic Cancer

**DOI:** 10.3390/cancers15245708

**Published:** 2023-12-05

**Authors:** Sherise Rogers, Angel Charles, Ryan M. Thomas

**Affiliations:** 1 Department of Medicine, Division of Hematology and Oncology, University of Florida College of Medicine, Gainesville, FL 32610, USA; sherise.rogers@medicine.ufl.edu; 2 Department of Surgery, University of Florida College of Medicine, Gainesville, FL 32610, USA; angel.charles@surgery.ufl.edu; 3 Department of Molecular Genetics and Microbiology, University of Florida College of Medicine, Gainesville, FL 32603, USA

**Keywords:** pancreatic cancer, pancreatic ductal adenocarcinoma, microbiome, microbiota, immunotherapy

## Abstract

**Simple Summary:**

Pancreatic cancer is an insidious disease, often diagnosed late in its course when curative treatments are moot, with a 5-year median overall survival of ~12%. While other malignancies have recently shown a response to immunotherapy, targeting the immune system in pancreatic cancer has historically not been successful. Resistance to immunotherapy in pancreatic cancer is believed to be partly due to the immune-suppressive tumor microenvironment. The microbiome, comprised of trillions of microorganisms living in a pathobiont and symbiotic relationship with its host, has recently been demonstrated to modulate the efficacy of immunotherapy in several human cancers. This review focuses on the known interactions of the microbiome and its potential to mitigate pathways for immunotherapy resistance in pancreatic cancer, a deadly disease with limited treatment options.

**Abstract:**

Pancreatic ductal adenocarcinoma cancer (PDAC) is projected to become the second leading cause of cancer-related death in the United States by 2030. Patients are often diagnosed with advanced disease, which explains the dismal 5-year median overall survival rate of ~12%. Immunotherapy has been successful in improving outcomes in the past decade for a variety of malignancies, including gastrointestinal cancers. However, PDAC is historically an immunologically “cold” tumor, one with an immunosuppressive environment and with restricted entry of immune cells that have limited the success of immunotherapy in these tumors. The microbiome, the intricate community of microorganisms present on and within humans, has been shown to contribute to many cancers, including PDAC. Recently, its role in tumor immunology and response to immunotherapy has generated much interest. Herein, the current state of the interaction of the microbiome and immunotherapy in PDAC is discussed with a focus on needed areas of study in order to harness the immune system to combat pancreatic cancer.

## 1. Introduction

Pancreatic ductal adenocarcinoma (PDAC) is the most common malignancy of the pancreas as it accounts for >90% of all pancreatic tumors. It is currently the third leading cause of cancer-related death in the United States but is predicted to overtake colorectal cancer as early as 2030 to become the second leading cause [1,2,3]. While a discrete inciting event has not been identified and most cases are spontaneous, risk factors include advanced age, tobacco smoking, chronic pancreatitis, diabetes mellitus, obesity, ethnicity, and family history. While curative surgical resection is the only possibility for cure, the use of ancillary treatment in the preoperative, and postoperative setting maximizes the possibility of long-term survival. However, PDAC is notorious for a tumor microenvironment (TME) that is composed of immune cells, fibroblasts, extracellular matrix, and endothelial cells. While each component has been shown to play a role in the immunosuppressive environment in PDAC, a detailed discussion is outside of the scope of this manuscript, with excellent reviews having already been published on this topic [4,5,6]. This cellular network has been shown to create an immunosuppressive environment that limits the host response of tumor detection and destruction [7]. It would therefore be natural to investigate ways to modulate the cellular responses in the TME for beneficial effect. However, clinical trials aimed at altering the TME to allow immune cell activation have been disappointing [8,9]. Regardless, harnessing the host immune system for cancer treatment is an extremely promising field that has already led to many advances. How pancreatic cancer fits into this area is still yet to be determined, but the identification of novel factors that alter the response to immunotherapy in PDAC is still desperately needed.

The ability of PDAC to create an immunosuppressive TME has been linked to interactions between the host microbiota and the innate and adaptive immune cells [10]. The microbiota, the collection of microorganisms including bacteria, viruses, fungi, and archaea that live on and within every human, has been shown to play an essential role in physiologic homeostasis. Carcinogenesis has been shown to be influenced by the direct impact of bacterial toxins/metabolites on cancer and immune cells, regulation of the local and systemic immune response, and alterations in metabolism [11]. In PDAC, patients with long-term survival were found to have a higher alpha-diversity in the tumor microbiome than short-term survivors [12]. Fecal microbiota transplant from these long-term survivors into mice bearing PDAC tumors led to changes in the tumor microbiota, reduced tumor growth, and the ability of immune cells to invade the TME compared to fecal microbiota transplant from short-term survivors. These studies suggest that the TME cross-talks with the gut microbiome and influences the host immune response to the tumor. Multi-cohort studies have also provided evidence for a potential “microbial signature” associated with PDAC [13,14]. These source microbiotas were both oral and intestinal (fecal), with prior studies demonstrating the impact of the fecal and oral microbiomes not only on pancreatic carcinogenesis but also on immune modulation in PDAC [15,16,17]. Exploiting the influence of the host microbiota over the immune landscape has become a potential avenue for the creation of novel therapeutics and may improve the efficacy of existing immunotherapies in the treatment of PDAC.

Despite extensive research on the molecular pathogenesis of pancreatic ductal adenocarcinoma, exciting discoveries identifying which cellular pathways are altered in PDAC development, progression, and metastasis, and identification of patterns in the microbiome profiles of patients with PDAC, there has been limited success clinically, and only marginal improvement in the morbidity and mortality of the disease. Failure to improve survival in patients with PDAC is thought to be related to a limited understanding of the relationship between the PDAC TME, the gut microbiome, and the immune system. Herein, we provide a general overview of what is known regarding the influence of the microbiome on immune function in PDAC and discuss the potentials and pitfalls in improving PDAC response to immunotherapies through modulation of the microbiome.

## 2. The Prospect of Immunotherapy in Pancreatic Cancer Treatment

Immunotherapy can be classified into four main categories: cytokines, vaccines, cellular therapies, and antibodies (among which immune checkpoint inhibitors are included) [18,19]. In general, the primary goal of immunotherapy is to activate the immune system to facilitate cancer surveillance and killing, which is an active area of laboratory research in PDAC (Table 1). Cytokine therapies were one of the earliest forms of immunotherapy used in the treatment of malignancies, with interleukin-2 (IL-2) and interferon alpha (IFN-α) serving as the hallmarks of this treatment modality. Cytokine treatments, such as these, used contemporary immunology knowledge of the time with the aim to activate the immune system to target and kill cancer cells. Such treatment is not specific and resulted in off-target effects resulting in systemic inflammatory activation and patient morbidity. For example, treatment with IL-2 or IFN-α serves to increase T-cell and NK-cell expansion and activation, and both are FDA-approved for melanoma. A modest survival benefit was incurred, but the treatment attrition was high because of side effects (10–37%) [20,21]. Given its poor tolerance and lack of effectiveness in PDAC, this is not an approved therapy for this malignancy. With advancing technology and knowledge, more targeted approaches that engage the immune system, as described below, have subsequently been developed.

**Table 1:** Selected preclinical studies of immunotherapy in PDAC.

Author	Immunotherapy Category	Result
Zhao [22]	ICI	Enhanced immunotherapy response after IL2-inducible T-cell kinase inhibition.
Hung [23]	CAR-T	Protease-activated receptor 1 CAR-T cells enhanced PDAC xenograft killing and decreased matrix metalloproteinase 1 levels in the TME.
Xu [24]	ICI BiTE	Conversion of immunosuppressive Tregs to immune-enhancing Tregs.
Beelen [25]	ICI	Increased NK-cell-induced PDAC organoid cell death with anti-PD-L1 or anti-HER2 therapy.
Qiang [26]	Hu-mAb	TGF-β blockade in orthotopic PDAC xenografts enhances sensitivity to chemotherapy.
Koh [27]	Immunostimulatory	Ex vivo activated NK cells inhibit PDAC xenograft growth in combination with gemcitabine.
Li [28]	CAR-T	CAR-T cells targeting glypican-1 resulted in PDAC xenograft regression and increased T-cell signaling.
Peng [29]	ICI Vaccine	ICI reverses T-cell dysfunction and enhances neoantigen vaccine-induced T-cell responses and PDAC xenograft regression.
Mirji [30]	ICI	Enhanced immune activation and PDAC xenograft response to ICI with the microbial metabolite trimethylamine N-oxide.
Pushalkar [10]	ICI	Depletion of microbiota increases PD-1 expression and efficacy of anti-PD-1 ICI.
Winograd [31]	ICI	ICI resistance can be overcome with the induction of T-cell immunity.
Luu [32]	CAR-T	Microbial-derived short-chain fatty acids enhance antitumor immunity against PDAC xenografts.

Immune checkpoint inhibitor (ICI); chimeric antigen receptor-modified T cells (CAR-T cells); pancreatic ductal adenocarcinoma (PDAC); tumor microenvironment (TME); bispecific T-cell engager (BiTE); Humanized Monoclonal Antibody (Hu-mAb).

Therapeutic vaccines function by enhancing the activation of tumor-specific B or T cells as well as upregulating tumor antigen presentation. Sipuleucel-T is an autologous vaccine that is FDA-approved for castrate-resistant prostate cancer [33]. The Bacillus Calmette–Guérin (BCG) vaccine is a live, attenuated form of *Mycobacterium bovis*. While it was initially utilized for immunization against tuberculosis, it is commonly used in the treatment of superficial bladder cancer through immune activation [34,35]. However, in pancreatic cancer, several vaccine clinical trials have been attempted without success, while others are still in the early phase of testing [36]. These vaccines can target tumor-associated or specific antigens, each of which poses different challenges [37]. Tumor-associated antigens have the potential to non-discriminately target host cells, while tumor-specific antigens are dependent upon a high mutational burden for effectiveness that is not commonly present in PDAC [38]. Furthermore, the intratumoral heterogeneity poses a challenge to PDAC vaccine development in which tumor-associated antigen targeting would be useful. Nevertheless, messenger RNA (mRNA) vaccines show promise in pancreatic cancer as a personalized therapy by encoding customized proteins based on the genetic profile of an individual’s tumors, specifically mutant Kras [36,39]. Additionally, a recent phase I trial evaluated the efficacy of personalized RNA neoantigen vaccines. Using surgically resected specimens, the investigators synthesized mRNA neoantigen vaccines in real time. Patients were subsequently treated with their personalized vaccine, targeting up to 20 major histocompatibility complex class I (MHC-I) and class II (MHC-II) restricted neoantigens after the administration of anti-PD-L1 immunotherapy (atezolizumab) but before systemic chemotherapy. This resulted in a robust expansion of neoantigen-specific T cells in 8 out of 16 patients. Those with vaccine-expanded T cells had a longer recurrence-free survival compared to those who did not have T-cell expansion [40]. This combinatorial treatment demonstrates the potential to delay PDAC recurrence, a common problem in patients who undergo potentially curative resection. Notably, the microbiota has been demonstrated to alter the MHC-II expression of intestinal epithelial cells and is responsible for discordant graft-versus-host disease in patients undergoing stem cell transplantation [41]. This raises the possibility that the microbiota may be able to alter MHC expression in PDAC patients, potentially providing vaccine targets.

Adoptive cell therapies are composed of T cells or NK cells which are either autologous or allogenic and specifically engineered to target specific proteins with a chimeric antigen receptor and T-cell receptors to recognize a peptide/MHC complex designed to ultimately kill cancer cells [37]. Several chimeric antigen receptor (CAR) T-cell therapies are approved for various hematological malignancies [42]. Tisagenlecleucel was the first of its kind and was approved by the FDA in 2017 for the treatment of pediatric and young adult acute lymphocytic leukemia. Unfortunately, limitations in homing, proliferation, and survival of transferred adoptive cells have limited its utility in solid tumors [43]. A possible way to circumvent this is the use of CRISPR/Cas9 technology for gene editing to increase the expression of transgenic receptors and deletion of inhibition signals. Several tumor-associated antigen adoptive cell therapies have failed due to their decreased specificity and increased toxicity. However, neoantigen epitope-specific CAR-T-cell targets are being tested and include mesothelin and KRAS (G12V mutation), which may show future promise and decreased toxicity.

Immune checkpoint inhibitors (ICIs) function by blockade of cell–cell interactions between host immune cells and target cells that serve to prevent immunologic activation [44]. While this is meant to be a tolerogenic process to prevent activation and killing of host cells, cancer has adapted features to likewise engage in these checkpoint inhibitory processes, render immune detection and activation of cancer cells inert, and thus functionally avoid detection by the immune system. With the identification of the cytotoxic T-lymphocyte associated protein 4 (CTLA4), programmed cell death protein-1 (PD-1), and PD-1’s ligand (programmed cell death ligand 1; PD-L1), the possibility of targeted immune therapy became a reality [44]. This important field was recognized with the Nobel Prize in Physiology or Medicine in 2018 for the “discovery of cancer therapy by inhibition of negative immune regulation” [45]. This notoriety came partly from several phase 3 clinical trials of ICIs that demonstrated durable treatment responses with prolonged recurrence-free survival (RFS) and overall survival (OS), even in the metastatic setting. For example, melanoma and lung cancer were shown to be sensitive to CTLA-4 and PD-1/PD-L1 blockade with improvements in RFS and OS [46,47,48,49]. Several gastrointestinal malignancies have also been shown to be responsive to the inhibition of these pathways, including hepatocellular cancer [50], gastric cancer, and esophageal cancer [51,52]. While it is natural to apply these various immunotherapies to PDAC treatment, unfortunately, none of these immunotherapy classes have proven to be successful in clinical trials to date, but studies are ongoing (Table 2). Additionally, several monoclonal antibodies (mAbs) have been studied as a therapeutic target for pancreatic cancer independent of immune checkpoint inhibition [53]. In recent years, there has been an increase in the number of mAbs that are FDA-approved for cancer. Monoclonal antibody therapy can be tailored based on the antibody format (full chain, fragment, variable heavy or light chains) which dictates specificity, half-life, and tissue penetration [54]. Besides direct pathway inhibition (as seen with anti-PD-1/PD-L1 therapy), antibodies can also be conjugated to drugs or radionucleotides for payload delivery directly to a tumor [55,56]. The most recent and notable example of antibody use to treat PDAC is the use of olaparib, a PARP inhibitor that prevents the repair of single-strand DNA breaks and leads to the accumulation of double-strand DNA breaks and cell death, in patients with metastatic PDAC and a germline mutation of the *BRCA* gene, which normally encodes proteins responsible for homologous recombination of double-stranded DNA breaks [57]. The use of this antibody therapy resulted in a prolonged progression-free survival in patients treated with olaparib compared to control (HR 0.53, 95% confidence interval 0.35–0.82, *p* = 0.004). To date, this mAb therapy has been the exception, rather than the rule, for PDAC treatment. Understanding the resistance mechanisms of PDAC in response to these various immunotherapies is therefore imperative to improve treatment options for patients with this deadly disease.

Although data support a survival advantage in patients with increased intratumoral immune infiltration in PDAC and that chemotherapeutic regimens can induce beneficial immunologic changes in the PDAC tumor microenvironment (TME) [58,59], achieving antitumor immune cell infiltration for tumor control/treatment has remained elusive. Specifically, the PDAC TME comprises a variety of cells that create an immunosuppressive environment as described previously [60]. Some of these cells, such as pancreatic stellate cells, function to sequester and inhibit infiltration of T lymphocytes [61]. The spatial relationship of the immune infiltration may also prevent response to treatment, such as neoadjuvant chemoradiation [62]. Additionally, the PDAC TME functions as a physical barrier through its carcinoma-associated fibroblasts, endothelial cells, and hyaluronic acid deposition that is thought to inhibit the penetration of systemic therapies. Several trials have aimed to alter the stromal composition of the PDAC TME in an effort to improve therapy delivery [63,64], with disappointing results [8,9,65]. Identifying unique host factors that can modulate immune infiltration and activation in PDAC may lie in the host microbiome, which has demonstrated an impact on immunotherapeutic efficacy in several cancers [66,67,68].

## 3. Overview of Microbiome–PDAC Research

Considerable progress has been made in our knowledge of the influence of the microbiome on pancreatic carcinogenesis since the initial studies demonstrating associations between the fecal and oral microbiome and pancreatic cancer [17,69,70]. Subsequently, preclinical data have demonstrated that the presence of an intact microbiome can accelerate pancreatic carcinogenesis in xenograft and genetic mouse models of pancreatic cancer [71,72]. In these studies, both antibiotic depletion of intestinal bacteria and germ-free mice born and raised in an environment void of microbes were utilized. Immune profiling of these tumors demonstrated reduced immune infiltration in mice harboring an intact microbiome. Specifically, Pushalkar et al. provided evidence that microbiota ablation resulted in a decrease in myeloid-derived suppressor cells (MDSCs) with a concomitant increase in M1-polarized (antitumor) macrophage differentiation which subsequently promoted Th1 differentiation of CD4+ lymphocytes and CD8+ lymphocyte activation [10]. Members of our group reported a similar reduction in immune cell infiltration but utilized a Nod/SCID mouse model, lacking a competent immune system [71]. This study suggests that the microbiome can modulate the antitumor function of both the adaptive and innate immune systems. In a follow-up study, the anti-PDAC activity of natural killer (NK) cells was specifically noted to be altered by the status of the host microbiome, with an intact microbiome reducing NK-cell infiltration and activation in PDAC xenografts [73]. These studies provide evidence of a biologic interaction of the microbiome with pancreatic carcinogenesis, later supported by human correlative studies.

**Figure 1:**
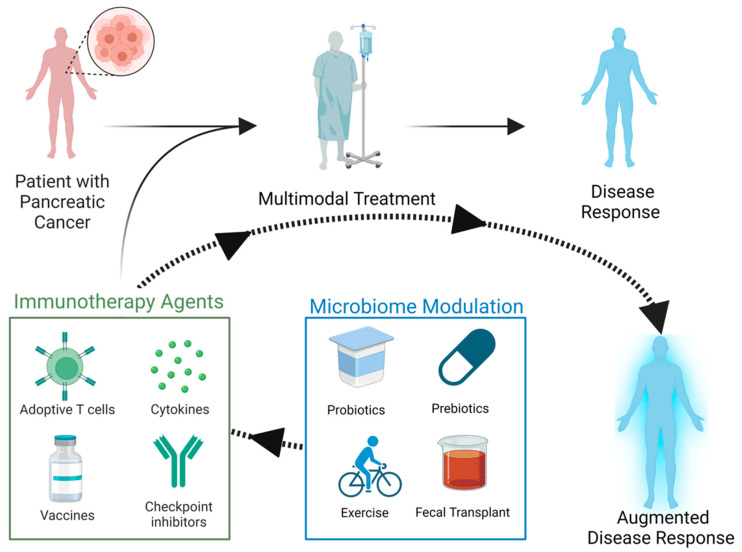
Potential elements of immunotherapy and microbiome manipulation to augment the response of pancreatic cancer to immunotherapy. Figure created with Biorender.com (accessed on 17 October 2023).

As an extension to these preclinical studies, several groundbreaking human studies have yielded important insight into the role of the microbiome in PDAC, solidifying this important area of research. As previously mentioned, the intratumoral microbiome was characterized in patients deemed “long-term survivors” (LTSs) and “short-term survivors” (STSs) [12]. Patients in the LTS cohort had increased microbial diversity compared to STSs, and their tumors demonstrated a more “immune-activated” environment with increased CD3 and CD8 cell infiltration and granzyme B(+) cells, a marker of activation. Furthermore, this immune profile in the LTS patients was correlated with the presence of the genera *Saccharopolyspora*, *Pseudoxanthomonas*, and *Streptomyces* in the LTS PDAC tumors, suggesting that bacteria from these genera may facilitate immune cell recruitment and activation. Finally, a recent study characterized the fecal microbiome of 57 Spanish patients with PDAC, 50 healthy controls, and 29 patients with chronic pancreatitis in a discovery phase and 76 German PDAC patients in a validation phase to assess the ability to diagnose PDAC [14]. Based on a 27-species panel, the accuracy of diagnosing PDAC, independent of stage, was 84%, and with the addition of CA19-9 as either normal or elevated, the accuracy of diagnosis increased to 94%. This provides hope for early detection techniques in high-risk individuals but also affirmation for microbiome-focused clinical trials in pancreatic cancer. While many immunotherapy trials in PDAC have not proven clearly effective to date, identifying and modulating novel factors that may limit or enhance the effectiveness of immunotherapy are important areas of investigation (Figure 1).

## 4. The Relationship of the Microbiome with Conventional Chemotherapy in PDAC

Systemic chemotherapy has been the backbone for the treatment of PDAC since the discovery of 5-fluorouracil (5-FU) and its associated survival benefit when used in the treatment of this disease [74,75,76,77]. Since then, a variety of regimens and treatment schedules have been investigated in both the potentially curative and the palliative settings [75,78,79,80]. Despite changes in the chemotherapeutic strategy over the years, what has not changed, at least significantly, is the survival of PDAC patients treated with conventional systemic chemotherapy. It is for this reason that alternatives, such as immunotherapy, have been sought to improve the recurrence-free and overall survival rates of patients with PDAC [81]. In fact, many trials currently attempt to address that issue (Table 2). There are many reasons that may explain the low response rates of PDAC to systemic chemotherapy. Recently, the influence of the microbiome has become of interest [82,83] and may even provide insight or future direction to improve the responsiveness of PDAC to immunotherapy. As previously noted, a compositional difference in the intratumoral microbiota has been shown in long-term vs short-term survivors of PDAC after surgery and adjuvant systemic chemotherapy [12]. Whether this is merely an association or reason for the improved long-term response is unclear. However, ample evidence exists for bacterial-mediated alteration of chemotherapy efficacy through a variety of mechanisms [84,85]. One notable mechanism is the metabolism or inactivation of drugs. In fact, a recent study demonstrated that bacteria can metabolize gemcitabine, a common backbone of chemotherapy for PDAC, based on the length of their cytidine deaminase enzyme isoform [86]. Metabolism and inactivation of chemotherapy are not the only potential deleterious actions on chemotherapy. Activation of a once inactive compound can be problematic as well from a toxicity standpoint. For example, bacteria have been shown to reactivate irinotecan, a component of the folinic acid, fluorouracil, irinotecan, and oxaliplatin (FOLFIRINOX) regimen used in PDAC, in the intestine after hepatic metabolism and biliary excretion [87]. This leads to the reactivation of the inactive compound in the gut which can have the dose-limiting adverse effect of colitis and associated diarrhea. How the microbiome modulates chemotherapeutic efficacy is an understudied area, and trials that include traditional systemic chemotherapy with immunotherapy (Table 2) will need to eventually account for the influence of the microbiota on individual and combined components of each treatment regimen.

## 5. Overcoming Challenges in PDAC Immunotherapy with the Microbiome

**Figure 2:**
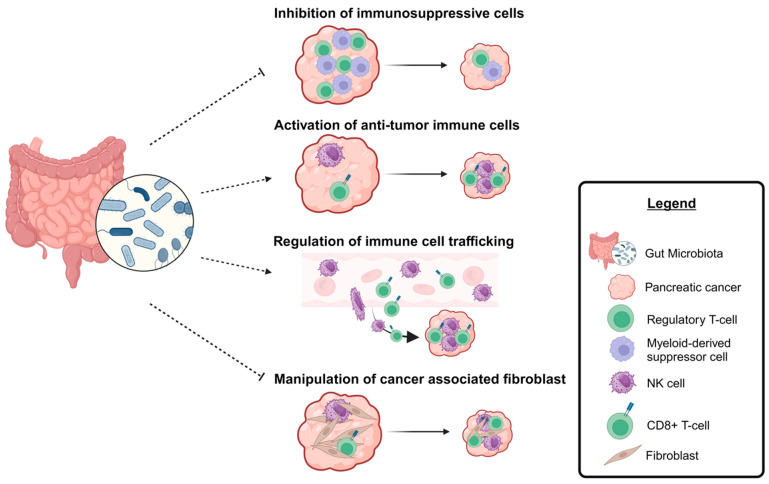
Microbiome-related strategies to improve the response of PDAC to immunotherapy. Figure created with Biorender.com (accessed on 17 October 2023).

It is well documented that the PDAC TME is an immune-privileged location that possesses immunosuppressive properties [6,88]. If microbiome manipulation is to be used to regulate this environment, several candidate areas will need to be targeted for investigation (Figure 2). While it is unknown whether targeting the pancreatic or gut microbiota will yield the most promising results, the subsequent discussion assumes the gut microbiome is manipulated through the presented modalities (Figure 2). While the pancreatic microbiota has been modulated experimentally through oral gavage [10], these manipulations were performed with supra-physiologic amounts of bacteria that likely do not have physiologic relevance but do demonstrate a proof of concept. From a therapeutic standpoint, the introduction of beneficial bacteria is an easier task than the elimination of specific deleterious bacteria, but this simplistic view does not account for the community structure of the microbiome and bacteria–bacteria interactions. Furthermore, there is no direct way to manipulate the intrapancreatic microbiota at present, although bacteriophage therapy to target specific culprit species is an active area of investigation.

Given this, the inhibition of immune-suppressive cells may be central to this intervention given the stronghold that these cells have on PDAC regulation, and this is the first area of discussion. Typical immune-suppressive cells in the TME include myeloid-derived suppressor cells (MDSCs), macrophages (specifically M2 polarized macrophages), and regulatory T cells (Tregs) [89,90]. Prior data demonstrate that the gut microbiome can promote MDSC accumulation in the liver and promotion of cholangiocarcinoma, an upper gastrointestinal malignancy with similarities to PDAC [91]. In this study by Zhang and colleagues, inflammatory disruption of the gut barrier allowed the translocation of bacteria to the liver, which induced CXCL1 expression in hepatocytes via a toll-like receptor-4 (TLR-4)-dependent manner and the accumulation of polymorphonuclear myeloid-derived suppressor cells. Treatment with antibiotics (neomycin) reversed these effects and inhibited tumor growth. Pushalkar and colleagues additionally demonstrated that the microbiome can promote pancreatic carcinogenesis by inducing immunosuppressive properties of the adaptive and innate immune systems, also through activation of toll pathways [10]. Finally, much effort has recently been devoted to the effect of microbial-derived metabolites/byproducts on immune cell modulation. Tumor-associated macrophages (TAMs) are a critical component of the PDAC immune microenvironment. Their polarization into M1 versus M2 macrophages dictates the antitumor properties that they possess, with M2 TAMs commonly associated with PDAC and immune suppression [92,93]. The aryl hydrocarbon receptor (AhR) binds indoles, products of tryptophan metabolism by bacteria and suppressors of immune inflammation. Upon binding, the AhR is translocated into the nucleus where downstream regulation of immune cells, including macrophages, occurs. Hezaveh and colleagues demonstrated that dietary tryptophan is metabolized by the gut microbiota into indole compounds that subsequently activate TAM AhR to inhibit the activation and antitumor function of CD8 T cells, as evidenced by decreased interferon gamma expression [94]. The state of the TAM AhR dictated the immunologic profile of the PDAC microenvironment, and inhibition of the AhR promoted inflammation within PDAC tumors and, notably, response to anti-PD-L1 treatment. In this manner not only could dietary manipulation alter the impact of the microbiome on the PDAC immune environment, but AhR inhibition may be an avenue for limiting the immune-suppressive environment of PDAC tumors.

Second, activation of the antitumor immune compartment will be an important component of harnessing the microbiome to improve the response of PDAC to immunotherapy. It has been shown that distinct microbiota members are associated with treatment response to immunotherapy in melanoma and that fecal transplantation can promote response to such therapy [95,96]. This activation is thought to be mediated by immune cell activation. For example, Uribe-Herranz and colleagues reported that the efficacy of adoptive T-cell therapy is dependent on the state of the microbiome, with antibiotic elimination of specific bacterial classes leading to an increase in CD8-alpha dendritic cells that supported the adoptively transferred antitumor T cells in an IL-12-dependent manner [97]. Further evidence for the importance of dendritic cells (DCs) and microbiome-associated immune cell activation was provided by Tanoue et al. when it was demonstrated that a consortium of 11 human-derived fecal bacteria was able to induce the antitumor phenotype in CD8 T cells in the intestine via interferon gamma (IFNg) production [98]. This was dependent on CD103+ DCs and major histocompatibility (MHC) 1a molecules. Notably, a recent study by Mirji and colleagues identified the bacterial metabolite trimethylamine N-oxide (TMAO) as a potent modulator of antitumor immunity specifically in PDAC [30]. The mechanism was through the activation of type-I interferon gamma pathways which activated antitumor macrophages and effector T cells in the tumor microenvironment. Supplementation of mice bearing PDAC xenografts with TMAO or dietary choline (a TMAO precursor) resulted in smaller xenografts. Additionally, co-treatment with TMAO and an ICI resulted in a synergistic tumor reduction in tumor burden compared to that recorded for any one treatment alone. This represents an opportunity not only to activate antitumor immunity in the host, but also to activate antitumor immunity from within the intestines, which could have a great systemic impact.

A third area of research that must be targeted to potentially enhance the responsiveness of PDAC to immunotherapy in a microbiome-mediated fashion is through the regulation of immune cell trafficking. The intratumoral microbiome has been demonstrated to regulate CD8 T-cell infiltration in melanoma patients, which has a direct impact on survival [99]. While melanoma is an immunogenic cancer, given the dense, fibrotic nature of PDAC tumors, this presents a challenge in these patients. However, the pancreatic TME indeed comprises immune cells, demonstrating the ability for infiltration. Members of our group have demonstrated that the state of the microbiome and microbe-derived products are able to influence the infiltration and activation of antitumor NK cells in PDAC tumors in vivo [73]. Additionally, using a computational pipeline to evaluate single-cell sequencing from two PDAC cohorts, bacteria were identified that paired with host cells in PDAC but not non-malignant tissue [100]. This pairing was associated with not only the activation of immune signaling pathways but also cell motility. The interaction of the tumor-associated microbiome may therefore be capable of modulating immune cell trafficking and provides an entry point for pharmaceutical targeting. Finally, in a murine pancreatitis model, fecal transplantation from chronic pancreatitis mice into healthy donor mice exacerbated pancreatic fibrosis, CD4 T-cell infiltration, and macrophage infiltration, potentiators of immune suppression, although macrophage polarization was not reported. These examples demonstrate a potential bidirectional influence of the microbiome for pro- and antitumor immune infiltration into the pancreas. The modulation of immune infiltration into PDAC that can subsequently be activated through microbiome manipulation is thus a very enticing concept for improving responsiveness to immunotherapy.

Finally, and common for PDAC, is the role of cancer-associated fibroblasts (CAFs) and whether their role can be manipulated via the microbiome to increase responsiveness to immunotherapy. Cancer-associated fibroblasts impart the desmoplastic structure to PDAC and are thought to pose a barrier to immune infiltration and drug delivery. While prior clinical trials proved to be unsuccessful in remodeling the PDAC TME to improve drug delivery and response [9], there is still much interest in this field. Interestingly, PDAC fibroblasts produce homotrimeric collagen as opposed to normal heterotrimeric collagen. This homotrimeric collagen promotes tumorigenic signaling and chemotherapy resistance and is associated with the order Bacteroidales within the TME. The deletion of the homotrimers increases T-cell infiltration and the responsiveness to anti-PD-1 immunotherapy [101]. Data are otherwise limited in this realm, and additional investigations are required to determine if the microbiome can elicit TME remodeling that will enhance immunotherapy effectiveness.

## 6. Conclusions

In order to advance our knowledge of the microbiome and its interaction with immunotherapy for PDAC, clinical trials are critical. To that end, there are active trials to test the efficacy of immunotherapy and microbiome manipulation in pancreatic cancer (Table 3). While trials with associative endpoints of microbiome analysis have been performed or are underway as it relates to immunotherapy [81,102,103], it is readily apparent that trials testing actual microbiome modulation and its effect on immunotherapy are sparse. Given prior studies delineating the role of the microbiome in immunotherapy efficacy as well as adverse events [96,104,105], such trials may yield important information for the relative insensitivity of PDAC to immunotherapy, but more are desperately needed. Additionally, the impact of nutrition on immunotherapy efficacy [106,107,108], as well as on microbiome diversity and composition [109,110,111], is well known, and investigators have taken this into account with recent trial designs to address the relationship between the three. Furthermore, various methods of exercise are known to alter microbiome diversity [112,113]. It can thus be hypothesized that such an intervention (nutrition or exercise) can alter immunotherapy response through microbiota modulation, and exciting data already exist for this [114,115]. There is a need for the design of trials that serve to determine the role of exercise and/or nutrition in modulating the microbiome and subsequent response to immunotherapy (Figure 1).

The frustratingly low survival of patients diagnosed with PDAC is mirrored by the continued difficulty in finding durable treatment regimens. While progress has been made, notably with the emerging data on the microbiome and PDAC progression, an increased understanding of the mechanisms that regulate the immune TME of PDAC is needed. Through clinical trials and a deeper understanding of microbial interactions with tumor signaling, the microbiome may prove to be the missing link for harnessing the power of immunotherapy in pancreatic cancer.

## Figures and Tables

**Table 2:** Active PDAC immunotherapy trials in the United States and Europe.

Study Title	Malignancy	Immunotherapy Category	Phase	ClinicalTrials.gov or EudraCT Study ID
Immunotherapy and Irreversible Electroporation in the Treatment of Advanced Pancreatic Adenocarcinoma	PDAC	ICI	II	NCT03080974
GVAX Pancreas Vaccine (With CY) in Combination with Nivolumab and SBRT for Patients with Borderline Resectable Pancreatic Cancer	PDAC	ICI Vaccine	II	NCT03161379
SD- 101, Nivolumab, and Radiation Therapy in Treating Patients with Chemotherapy Refractory Metastatic Pancreatic Cancer	mPDAC	ICI	I	NCT04050085
Pilot Study With CY, Pembrolizumab, GVAX, and IMC-CS4 (LY3022855) in Patients with Borderline Resectable Adenocarcinoma of the Pancreas	PDAC	ICI Vaccine	I	NCT03153410
Vaccine Therapy and Sargramostim in Treating Patients with Pancreas Cancer That Cannot Be Removed by Surgery	PDAC Pancreatic ACC	Vaccine	I	NCT00669734
Epacadostat, Pembrolizumab, and CRS-207, With or Without CY/GVAX Pancreas in Patients with Metastatic Pancreas Cancer	mPDAC	ICI Vaccine	II	NCT03006302
Testing the Combination of Anetumab Ravtansine with Either Nivolumab, Nivolumab and Ipilimumab, or Gemcitabine and Nivolumab in Advanced Pancreatic Cancer	PDAC	ICI	I/II	NCT03816358
Study of Pembrolizumab with or without Defactinib following Chemotherapy as a Neoadjuvant and Adjuvant Treatment for Resectable Pancreatic Ductal Adenocarcinoma	PDAC	ICI	II	NCT03727880
VX15/2503 and Immunotherapy in Resectable Pancreatic and Colon Cancer	CRC PDAC	ICI	I	NCT03373188
Neoadjuvant CAN-2409 in Combination with Chemoradiation or SBRT for Borderline Resectable Pancreatic Adenocarcinoma	PDAC	Viral immunotherapy	II	NCT02446093
Pancreatic Tumor Cell Vaccine (GVAX), Low Dose Cyclophosphamide, Fractionated SBRT, and FOLFIRINOX Chemotherapy in Patients with Resected Adenocarcinoma of the Pancreas	PDAC	Vaccine	I	NCT01595321
Study of CRS-207, Nivolumab, and Ipilimumab with or without GVAX Pancreas Vaccine (With CY) in Patients with Pancreatic Cancer	PDAC	ICI Vaccine	II	NCT03190265
A study of ELI-002 in Subjects with KRAS Mutated Pancreatic Ductal Adenocarcinoma (PDAC) and Other Solid Tumors	PDAC CRC NSCLC Ovarian cancer Cholangiocarcinoma Gallbladder carcinoma	Immunotherapy targeting KRAS mutants	I	NCT04853017
CAR T Cell Immunotherapy for Pancreatic Cancer	PDAC	CAR-T	I	NCT03323944
Study of Autologous T-cells in Patients with Metastatic Pancreatic Cancer	mPDAC	CAR-T	I	NCT03638193
Th-1 Dendritic Cell Immunotherapy Plus Standard Chemotherapy for Pancreatic Adenocarcinoma (DECIST)	PDAC	Vaccine	I	NCT04157127
Nivolumab and Ipilimumab and Radiation Therapy in MSS and MSI High Colorectal and Pancreatic Cancer	CRC PDAC	ICI	II	NCT03104439
Plerixafor and Cemiplimab in Metastatic Pancreatic Cancer	mPDAC	ICI	II	NCT04177810
An Open Label, Dose Escalation Followed by Dose Expansion, Safety and Tolerability Trial of CAN04, a Fully Humanized Monoclonal Antibody Against IL1RAP, in Subjects with Solid Malignant Tumors	NSCLC PDAC	Hu-mAb	I	2017-001111-36
A Phase Ib/II, Open-Label, Multicenter, Randomized Umbrella Study Evaluating the Efficacy and Safety of Multiple Immunotheraby-Based Treatment Combinations in Patients with Metastastic Pancreatic Adenocarcinoma (Morpheus-Pancreatic Cancer)	mPDAC	ICI Cytokine Inhibitor	Ib/II	2016-004126-42
Safety and Efficacy of IMM-101 Combined with Stereotactic Radiotherapy in Patients with Limited MEtastatic PANcreatic Cancer (MEPANC-1)	mPDAC	Immune Stimulatory	II	2020-003945-13
First-in-Human Study of ICT01 in Patients with Advanced Cancer (EVICTION)	PDAC Various other solid tumors	Hu-mAb Immune Stimulatory ICI	I/II	NCT04243499 2019-003847-31
Trial Of Hypofractionated Radiotherapy in Combination with MEDI4736 and Tremelimumab for Patients with Metastatic Melanoma and Lung, Breast and Pancreatic Cancers	PDAC NSCLC Melanoma	ICI	I	NCT02639026
Radiation Therapy in Combination with Durvalumab for People with Pancreatic Cancer	PDAC	ICI	I/II	NCT03245541

Pancreatic ductal adenocarcinoma (PDAC); immune checkpoint inhibitor (ICI); metastatic pancreatic ductal adenocarcinoma (mPDAC); colorectal cancer (CRC); non-small-cell lung carcinoma (NSCLC); acinar cell carcinoma (ACC); chimeric antigen receptor-modified T cells (CAR-T cells); humanized monoclonal antibody (Hu-mAb).

**Table 3:** Active microbiome-focused PDAC immunotherapy trials.

Study Title	Malignancy	Immunotherapy Category	Phase	ClinicalTrials.gov Study ID
Modulation of the Gut Microbiome with Pembrolizumab Following Chemotherapy in Resectable Pancreatic Cancer	PDAC	ICI	II	NCT05462496
ARGONAUT: Development and Analysis of a Blood and Stool Sample Bank for Cancer Patients, Enabling the Systemic Study of the Effect of Gut Microbiomes on Response to Treatment	PDAC CRC Triple Negative Breast Cancer NSCLC	ICI	Prospective Cohort Study	NCT04638751
The Mechanism of Enhancing the Antitumor Effects of CAR-T on PC by Gut Microbiota Regulation	PDAC	CAR-T	Case Control	NCT04203459 (China)
Feasibility Study of Microbial Ecosystem Therapeutics (MET-4) to Evaluate Effects of Fecal Microbiome in Patients on ImmunOtherapy (MET4-IO)	Solid Tumors	ICI	II/III	NCT03686202 (Canada)
Gut Microbiome Modulation to Enable Efficacy of Checkpoint-based Immunotherapy in Pancreatic Adenocarcinoma	PDAC	ICI	IV	NCT03891979 *

Pancreatic ductal adenocarcinoma (PDAC); immune checkpoint inhibitor (ICI); colorectal cancer (CRC); non-small-cell lung carcinoma (NSCLC); chimeric antigen receptor-modified T cells (CAR-T cells); trials are based in the United States unless otherwise specified. * Withdrawn.

## Data Availability

No new data were created for this literature review and discussion.
